# G protein-coupled receptors as candidates for modulation and activation of the chemical senses in decapod crustaceans

**DOI:** 10.1371/journal.pone.0252066

**Published:** 2021-06-04

**Authors:** Matthew T. Rump, Mihika T. Kozma, Shrikant D. Pawar, Charles D. Derby

**Affiliations:** 1 Neuroscience Institute, Georgia State University, Atlanta, Georgia, United States of America; 2 Yale Center for Genomic Analysis, Yale University, New Haven, Connecticut, United States of America; Wake Forest University, UNITED STATES

## Abstract

Many studies have characterized class A GPCRs in crustaceans; however, their expression in crustacean chemosensory organs has yet to be detailed. Class A GPCRs comprise several subclasses mediating diverse functions. In this study, using sequence homology, we classified all putative class A GPCRs in two chemosensory organs (antennular lateral flagellum [LF] and walking leg dactyls) and brain of four species of decapod crustaceans (Caribbean spiny lobster *Panulirus argus*, American lobster *Homarus americanus*, red-swamp crayfish *Procambarus clarkii*, and blue crab *Callinectes sapidus*). We identified 333 putative class A GPCRs– 83 from *P*. *argus*, 81 from *H*. *americanus*, 102 from *P*. *clarkii*, and 67 from *C*. *sapidus–*which belong to five distinct subclasses. The numbers of sequences for each subclass in the four decapod species are (in parentheses): opsins (19), small-molecule receptors including biogenic amine receptors (83), neuropeptide receptors (90), leucine-rich repeat-containing GPCRs (LGRs) (24), orphan receptors (117). Most class A GPCRs are predominately expressed in the brain; however, we identified multiple transcripts enriched in the LF and several in the dactyl. In total, we found 55 sequences with higher expression in the chemosensory organs relative to the brain across three decapod species. We also identified novel transcripts enriched in the LF including a metabotropic histamine receptor and numerous orphan receptors. Our work establishes expression patterns for class A GPCRs in the chemosensory organs of crustaceans, providing insight into molecular mechanisms mediating neurotransmission, neuromodulation, and possibly chemoreception.

## Introduction

The nervous system comprises a network of neurons that regulate the physiology and behavior of animals [1]. Neural networks are formed by distinct neuronal populations that regulate specific signaling pathways, while the intrinsic function of individual neurons is controlled by the proteins they express [2, 3]. For example, transmembrane receptor proteins act as internal and environmental sensors that transduce a received stimulus into a signal that can be transmitted throughout the nervous system to ultimately elicit a physiological or behavioral response [4]. Sensory receptors detect environmental stimuli in the form of light, sound, temperature, pressure, and chemicals, while internal receptors bind endogenous neurotransmitters and circulating hormones that mediate neurotransmission and signal modulation [5–8]. Prominent among the many types of transmembrane receptors are G protein-coupled receptors (GPCRs) [9–11]. In particular, class A (rhodopsin-like) GPCRs are the most abundant and diverse class of GPCRs among animals [12, 13], including in Pancrustacea, which includes insects and crustaceans [14–19].

Class A GPCR subclasses in crustaceans include opsins and receptors for small-molecule neurotransmitters, neuropeptides, and hormones. Most crustacean GPCRs that have been functionally characterized are involved in neurotransmission and neuromodulation rather than sensory transduction [14–19]. Opsins are an exception, being involved in photoreception in crustaceans as they are in other animals [20–22]. To date, GPCRs have not been shown to be chemoreceptors in crustaceans, but this issue has received little attention. This differs from the deuterostomes, where class A GPCRs are the dominant chemoreceptors [23–28]. Even in protostomes, which largely use ionotropic receptors as chemoreceptors, there are examples of GPCRs as chemoreceptors, including serpentine receptors in nematodes [29–32], opsins in *Drosophila* [33], and other GPCRs in gastropods [34, 35], ticks [36], and insects [37, 38].

Thus, our goal was to explore whether class A GPCRs are candidate chemoreceptors in crustaceans. To do this, we analyzed the transcriptomes of two chemosensory organs in four decapod crustacean species for all class A GPCRs. We also analyzed the brain transcriptomes from three of the four decapod crustacean species. The four species were Caribbean spiny lobster *Panulirus argus*, American lobster *Homarus americanus*, crayfish *Procambarus clarkii*, and blue crab *Callinectes sapidus*, chosen because they represent four of the major models of chemoreception in decapod crustaceans [39–41]. The two chemical sensing organs analyzed were the lateral flagellum (LF) of the antennule and the walking leg dactyls (S1 Fig). Both the LF and the dactyls have sensory neurons that are part of the distributed chemoreceptive system, but the LF solely constitutes the olfactory system through its olfactory sensory neurons (OSNs) in the aesthetasc sensilla (S1 Fig).

We identified class A GPCRs in the LF, dactyl, and brain transcriptomes, and we analyzed expression levels in all three organs. Among the GPCRs that we identified in our study, many have homologues that have been functionally characterized in other species including numerous small-molecule receptors, neuropeptide receptors, and hormone receptors. We believe that these receptors expressed in the chemosensory organs may be important for neurotransmission and neuromodulation involved with chemical sensing, especially GPCRs unique to the LF since it is the olfactory organ. In addition to the known types, we also identified several novel receptor types and multiple orphan receptors expressed in the chemosensory organs, which may also be involved in neurotransmission, neuromodulation, or the detection of external chemical cues.

## Materials and methods

### Ethics approval and consent to participate

Formal approval from the Institutional Animal Care and Use Committee of Georgia State University or other ethics committees was not required since our work did not involve vertebrate animals. Nonetheless, our protocols complied with standard practices including collecting organs and sacrificing animals using cold anesthesia.

### Organ collection, RNA sequencing, and *de novo* assembled transcriptomes

All analyses were performed on *de novo* transcriptomes that were previously generated by Kozma et al. 2020a [40]. Full details about animals, organ collection, RNA isolation, and RNA sequencing are described in Kozma et al. 2018 and 2020a [39, 40]. In brief, three organ-specific cDNA libraries were generated from *P*. *argus*, *H*. *americanus*, and *P*. *clarkii*: aesthetasc-bearing region of antennular lateral flagella (LF), sensilla-bearing dactyl of second walking legs (dactyl), and supraesophageal ganglion (brain) (S1 Fig). Two cDNA libraries were generated from LF and dactyl of *C*. *sapidus*.

For *P*. *argus*, organs collected from four adult animals (three females and one male): LF cDNA library was generated from pooled LFs from one male and one female; dactyl cDNA library was generated from one female; brain cDNA library was generated from one female. For *H*. *americanus*, organs were collected from one adult female and one adult male, and pooled for each organ type prior to RNA isolation. For *P*. *clarkii*, nine adult animals were used (five females and four males): LF cDNA library was generated from organs pooled from five animals; dactyl cDNA library was generated from pooled organs from one male and one female; and brain cDNA library was generated from pooled organs from one male and one female. For *C*. *sapidus*, ten adult animals (two females and eight males) were used to generate LF cDNA library, and organs from one female and one male were used to generate the dactyl cDNA library.

Quality assessment on Agilent Bioanalyzer2000 and TapeStation of total RNA extracted, mRNA specific cDNA synthesis, and cDNA paired-end sequencing on the Illumina HiSeq 2500 high-throughput sequencer were performed by Beckman Coulter Genomics (now part of GENEWIZ, South Plainfield, New Jersey) as previously described in Kozma et al. 2018 and 2020a [39, 40]. For *P*. *argus*, the read length was 2x100 (base pair reads) for LF and dactyl, and 2x125 (base pair reads) for brain. For *H*. *americanus*, the read length was 2x125 (base pair reads) for all three organs. For *P*. *clarkii*, the read length was 2x125 (base pair reads) for LF and dactyl and 2x100 (base pair reads) for brain. For *C*. *sapidus*, the read length was 2x125 (base pair reads) for both organs. The number of reads per sample was > 120 million. Adapter sequences tracking Illumina reads from multiplexed samples were removed prior to delivery.

Individual reference transcriptomes for *P*. *argus*, *H*. *americanus*, *P*. *clarkii*, and *C*. *sapidus* were previously developed by Kozma et al. (2020a) [40], which fully details the assembly process. In summary, a single non-redundant transcriptome for each species was created using the EvidentialGene (EVG) pipeline (https://f1000research.com/posters/5-1695). Raw reads were sequenced from the LF, dactyl, and brain cDNA libraries of *P*. *argus*, *H*. *americanus*, and *P*. *clarkii*, and from the LF and dactyl cDNA libraries of *C*. *sapidus*. All reads can be found under BioProject PRJNA59678 (https://www.ncbi.nlm.nih.gov/bioproject/prjna596786). EVG pipeline condenses multiple *de novo* assemblies, generated from the same set of raw reads by different assemblers, into one single refined transcriptome [42]. 3’ ends of raw reads were trimmed using Trinity-Trimmomatic v2.4.0 [43, 44]. Trinity v2.4.0 [43], Trans-ABySS v1.5.3 [45], Velvet v1.2.10 [46], and OASES v0.2.09 [47] assembly programs were used to generate eight *de novo* assemblies for each species. The following are the eight assemblies generated: Trinity v2.4.0 [43] was used to generate an unnormalized and a normalized *de novo* assemblies, normalization of reads was generated by default Trinity v2.4.0 settings. The remaining six assemblies were generated using normalized reads. The normalization of reads for these assemblies was done using FastUniq [48]. The third, fourth, and fifth *de novo* transcriptomes were generated using Trans-Abyss v1.5.3, with K-mer sizes 63, 87, and 111, respectively. The sixth, seventh, and eighth *de novo* transcriptomes were generated using Velvet v1.2.10 and OASES v0.2.09, with K-mer-sizes 63, 87, and 111, respectively. All eight assemblies were input into the EVG pipeline to generate a single refined and non-redundant transcriptome for each species. TransDecoder (http://transdecoder.github.io/) was used to predict protein sequences from EVG transcriptomes based on open reading frames (ORF). CD-Hit [49] was used on the predicted protein database to further remove redundancy, and the cd90 datasets generated by CD-Hit for each transcriptome were used for all further analyses. Throughout this manuscript, “transcript” refers to a contig (nucleotide) from the EVG transcriptomes and “sequence” refers to predicted protein sequence as generated by TransDecoder. To analyze the completeness of the assemblies, BUSCO v3 was run on all four EVG transcriptomes using the Arthropoda obd9 lineage (creation date: 2017-07-07, number of species: 60, number of BUSCOs: 1066). The full details of BUSCO analysis are available in Kozma et al. 2020a [40].

### Transcript abundance and differential expression

Transcript expression was determined by quantifying overall transcript abundance in each organ transcriptome and by comparing transcript expression levels between organ transcriptomes to find differentially expressed genes (DEGs). Transcript abundance was estimated using RSEM [50] in Trinity v2.8.2 [51] to generate a counts matrix of transcripts. DESeq2 [52] was used to find DEGs by calculating fold changes in transcript expression levels between organs using a custom ‘R’ script on the RSEM counts matrix files. RSEM counts matrices, DESeq2 analyses, and custom ‘R’ script are available from Kozma et al. (2020a) [40]. Here, we report the log_2_ [fold change] of class A GPCRs generated by DESeq2 between two organs of each species (S1–S4 Tables). From *P*. *argus*, *H*. *americanus*, and *P*. *clarkii*, LF vs. dactyl, LF vs. brain, and dactyl vs. brain were compared, and from *C*. *sapidus* LF vs. dactyl were compared. From the ten different DESeq2 comparisons for LF, dactyl and brain for each of the *P*. *argus*, *H*. *americanus*, *P*. *clarkii*, and *C*. *sapidus* species, MA-plots were generated on mean of normalized counts vs. fold changes (log_2_) from DESeq2 analyses (S2 Fig). Distribution of transcript IDs for the five different subclasses of class A GPCRs (opsin, small-molecule, neuropeptide, LGRs, and orphan) are colored on each of these ten MA-plots. DESeq2 data and custom ‘R’ code used to generate MA plots are available under accession S-BSST653 on EBI Biostudies (https://www.ebi.ac.uk/biostudies/studies/S-BSST653). We defined DEGs based on a log_2_ fold-change > 1.5 or log_2_ fold-change < -1.5; this metric has been deemed sufficient for determining DEGs in several studies [53–55]. We considered a log_2_ fold-change between -1.5 and 1.5 as no difference in expression, while a log_2_ fold-change greater than 1.5 or less than -1.5 indicates that there is a difference in expression between the two organ transcriptomes. A log_2_ fold change ± 1.5 is the equivalent of a ~2.82-fold change in expression; therefore, a fold change of 2.82 or greater was used to determine DEGs. We considered transcripts to be enriched in a particular organ if the fold-difference in expression exceeds 2.82 compared to another organ.

### GPCR identification, sequence alignment, and phylogenetic analysis

High performance computing systems at Georgia State University were used for this analysis [56, 57]. Identity of protein domain regions were predicted, from the EVG transcriptome cd90 predicted protein datasets, using InterProScan 5 v5.28–67.0 [58] by screening for Pfam conserved domains [59]. Class A GPCRs are distinguished by their seven-(pass)-transmembrane domain [60, 61] corresponding to the signature Pfam domain (PF00001). TMHMM v2.0. [62] was used to screen for transmembrane domains. Predicted protein sequences containing less than six predicted transmembrane helices were excluded from analyses, with the exception of four sequences from *C*. *sapidus* included in the opsin tree, which contained signature domain regions for light-sensitive opsin proteins. Reference protein sequences for characterized class A GPCRs were collected from various databases for select species and were used in phylogenetic analysis to determine sequence homology and classify the decapod sequences. Characterized class A sequences from *Drosophila melanogaster*, *Bombyx mori*, and *Caenorhabditis elegans* were obtained from FlyBase, SilkBase, and WormBase, respectively. Putative class A GPCRs from other decapod crustaceans were mined from published transcriptomes for the following species: *Cancer borealis*, *Carcinus maenas*, *Gecarcinus lateralis*, and *Nephrops norvegicus* [16–19]. Opsin sequences were obtained from multiple sources compiled by Porter et al. (2007) [21]. Indeterminate sequences were subject for BLASTP against NCBI non-redundant protein databases. Reciprocal BLAST matches representing characterized class A GPCR sequences demonstrating expected value (e value < 1 x e-5) were used as reference sequences. The aforementioned reference sequences and decapod sequences were compiled into individual query files (S1–S4 Files) as input for Jalview [63]. Sequences were aligned (S5–S8 Files) in Jalview using the MAFFT default setting [64, 65]. Following alignment, sequences were manually trimmed (S9–S12 Files) in Jalview to remove gaps and regions marked by low amino acid conservation (columns were trimmed if conservation score was below 0). Maximum likelihood phylogenetic trees were generated in IQ-Tree [66, 67] using 1000 ultrafast bootstrap [68] with ModelFinder [69] to generate confidence (bootstrap support) values and select the best model of substitution for the trees respectively. Phylogenetic trees were visualized in FigTree v1.4.4 to assess sequence homology and phylogenetic relationships between characterized reference sequence and decapod sequences. Homologues were in part implicitly determined based on the formation of clades containing decapod sequences and characterized reference sequences with the ultrafast bootstrap support (>89) at the outer nodes. Branch length and ultrafast bootstrap support at the inner nodes were also considered when defining homologues.

### GPCR nomenclature and classification

Sequences (and their corresponding transcripts) were named based on a two-part naming system comprising a prefix and suffix. The prefix abbreviation represents the respective decapod genus and species: Parg (*P*. *argus*), Hame (*H*. *americanus*), Pcla (*P*. *clarkii*), and Csap (*C*. *sapidus*). The suffix abbreviation represents the predicted receptor based on the homologue identified during phylogenetic analyses. Homology is based on sequence similarity to receptors characterized in past studies. These names are suggestive only, since we cannot yet confirm ligand sensitivity or functionality of these receptors.

For analyses yielding more than one homologue for a given receptor type, a number was assigned to each respective homologue (e.g., R1, R2) and a letter was assigned for multiple transcript variants (e.g., R1a, R1b). For example, three homologues were identified for the CCHamide receptor (e.g., CCHa-R1, CCHa-R2, CCHa-R3) and two variants for CCHaR1 in *P*. *argus* (e.g., CCHa-R1a, CCHa-R1b). Orphan sequences lacking a defined homologue were annotated as GPCR followed by an uppercase letter and a number to represent a cluster of sequences determined by phylogenetic analysis (e.g., Pcla_GPCR_A1, Pcla_GPCR_A2) and a lowercase letter to represent variants from the same species (Pcla_D2x, Pcla_D2y). Annotations for orphan receptors without homologues in other species are based on Fig 4, which only contains decapod sequences from our transcriptomes. Orphan receptors from *P*. *argus* were previously annotated by Kozma et al. (2020b) [41]; however; some of these annotations have been changed in this study (see S6 Table).

Sequences were also classified more generally according to the putative receptor subclass to which they belong. These categories include opsins, small-molecule receptors, neuropeptide receptors, leucine-rich repeat-containing GPCRs (LGRs, which are glycoprotein hormone receptors), and orphan receptors.

## Results

The total number of putative class A GPCR sequences containing the PF00001 domain and the number of sequences that met inclusion criteria for all four decapod species are shown in Table 1. In this table, the numbers of sequences containing the signature rhodopsin family domain (PF00001) for the four decapod crustaceans are listed in the first column, and the numbers of sequences meeting inclusion criteria (at least six predicted transmembrane helices) are listed in the second column.

**Table 1:** Number of sequences of class A GPCRs in crustacean transcriptomes.

Species	# of seqs with PF0001 domain	# of selected seqs
*Panulirus argus*	120	83
*Homarus americanus*	122	81
*Procambarus clarkii*	140	102
*Callinectes sapidus*	105	67

The numbers of sequences in each of the five subclasses of class A GPCRs–opsins, small-molecule receptors, neuropeptide receptors, leucine-rich repeat-containing GPCRs, and orphan receptors–are listed for each of the four decapod crustaceans in Table 2.

**Table 2:** Number of sequences in class A GPCR subclasses in crustacean transcriptomes.

Class A GPCR subclasses	Species			
	Parg	Hame	Pcla	Csap
Opsins	5	5	3	6
Small-molecule	22	22	23	16
Neuropeptide	23	19	34	14
LGRs	6	5	10	3
Orphans	27	30	32	28

**Fig 1:**
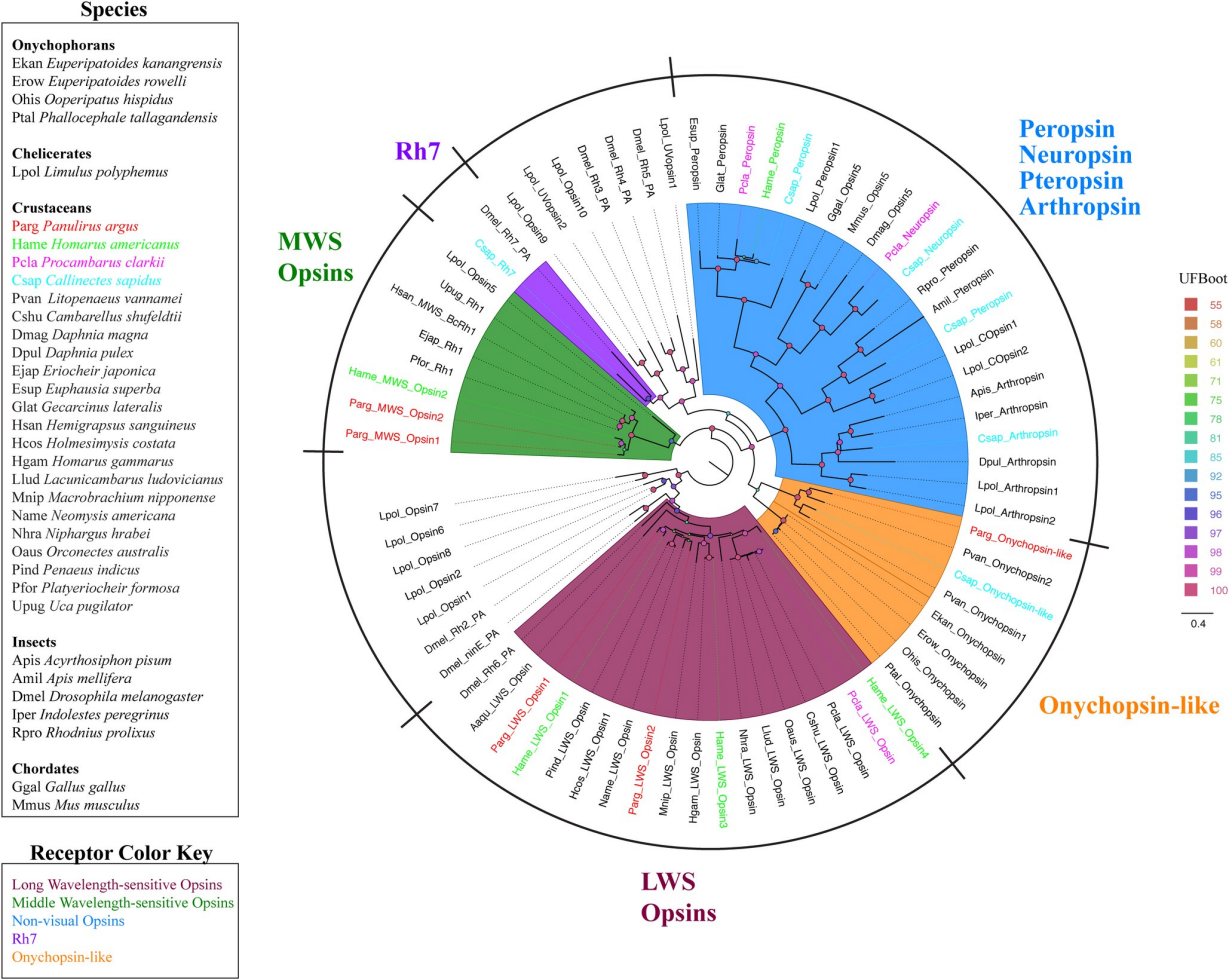
Maximum likelihood phylogenetic tree of opsins in four decapod crustaceans and other species. Colors represents five clades of visual and non-visual opsins. Maroon: Long wavelength-sensitive (LWS) opsins. Dark green: Medium wavelength-sensitive (MWS) opsins. Purple: *D*. *melanogaster* Rh7 homologues. Orange: Onychopsin-like molecules. Blue: Non-visual opsins including peropsins, pteropsins, neuropsins, and arthropsins. Colors representing decapod crustacean species: *P*. *argus* (red), *H*. *americanus* (green), *P*. *clarkii* (pink), *C*. *sapidus* (blue). The model of substitution is LG+F+I+G4 according to BIC as selected by ModelFinder. The root is drawn on the LWS opsin clade. Scale bar represents expected number of substitutions per site. Ultrafast bootstrap support values are color-coded and size-scaled from 55 to 100.

**Fig 2:**
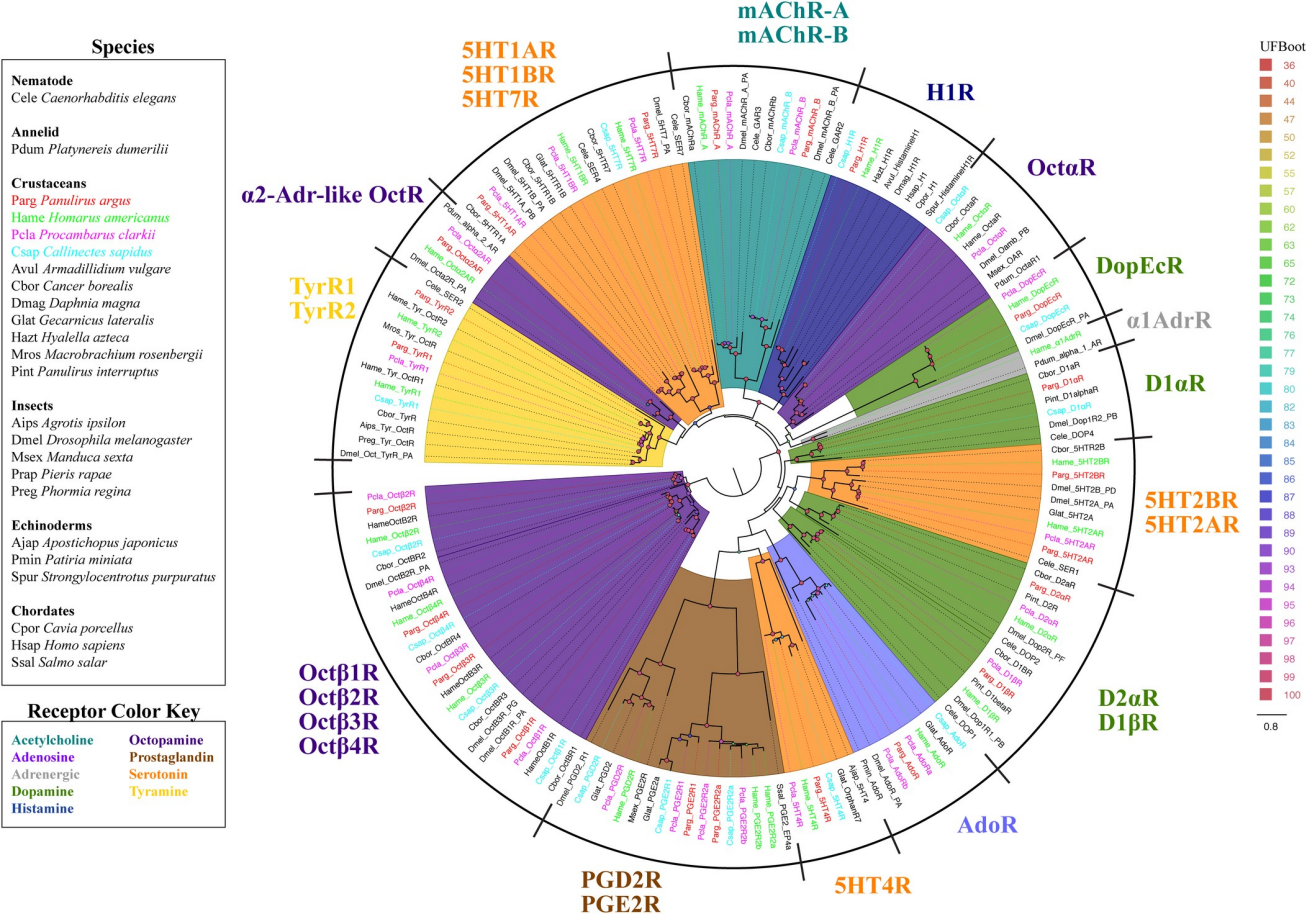
Maximum likelihood phylogenetic tree of small-molecule receptors in four decapod crustaceans and other species. Colors represent clades of sequences. Dark green: Dopamine and Dopamine/Ecdysteroid receptors; Orange: 5HT receptors; Teal: Acetylcholine receptors; Yellow: Tyramine receptors; Dark blue: Histamine receptors; Brown: Eicosanoid (prostaglandin) receptors (PGE); Light purple: Adenosine (Ado) receptors; Dark purple: Octopamine (Oct) receptors; Gray: Adrenergic receptors. Colors representing decapod crustacean species: *P*. *argus* (red), *H*. *americanus* (green), *P*. *clarkii* (pink), *C*. *sapidus* (blue). The model of substitution is LG+F+I+G4 according to BIC as selected by ModelFinder. Tree is unrooted and the root is drawn on the clade containing octopamine β receptors. Scale bar represents expected number of substitutions per site. Ultrafast bootstrap support values are color-coded and size-scaled from 36 to 100.

**Fig 3:**
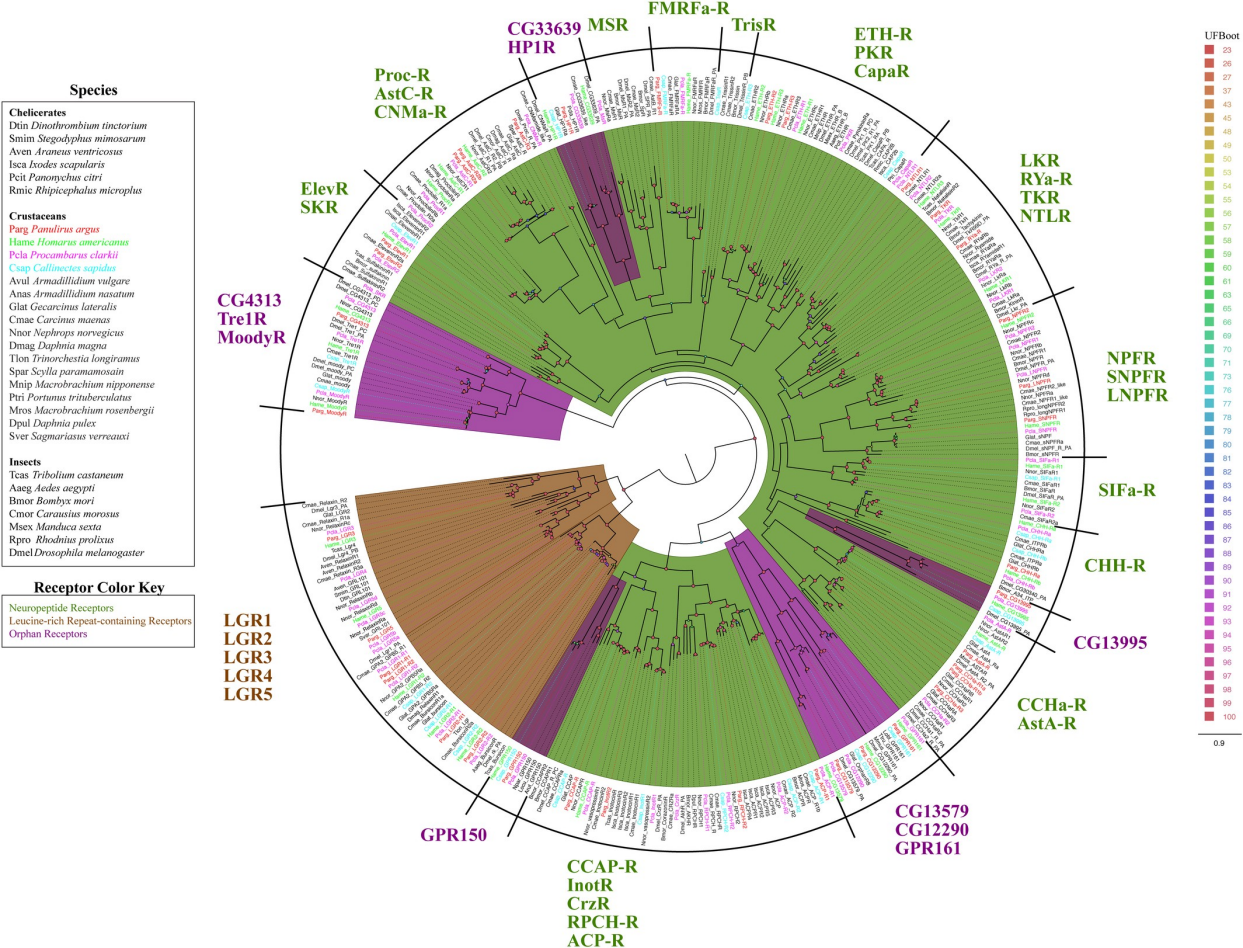
Maximum likelihood phylogenetic tree of neuropeptide receptors, leucine-rich repeat-containing GPCRs, and orphan receptors. Colors representing receptor types are neuropeptide receptors (dark green), leucine-rich repeat-containing GPCRs (brown), and orphan receptors (purple). Colors representing decapod crustacean species: *P*. *argus* (red), *H*. *americanus* (green), *P*. *clarkii* (pink), *C*. *sapidus* (blue). The model of substitution is LG+F+G4 according to BIC as selected by ModelFinder. Tree is unrooted and the root is drawn on the clade containing leucine-rich repeat-containing GPCRs. Scale bar represents expected number of substitutions per site. Ultrafast bootstrap support values are color-coded and size-scaled from 23 to 100.

**Fig 4:**
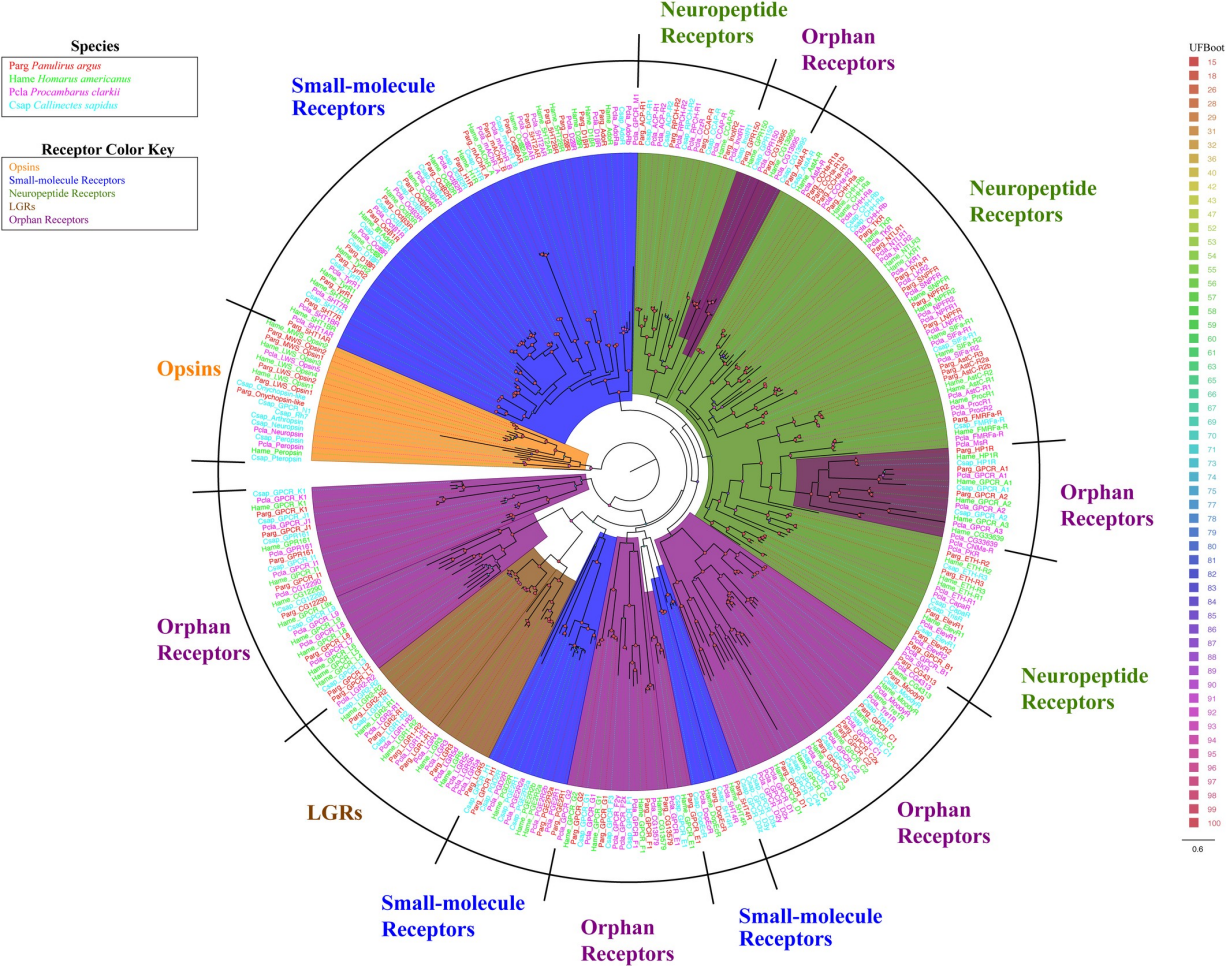
Maximum likelihood phylogenetic tree of sequences for all class A GPCRs in the four decapod crustacean species from this study. Colors representing the receptor clades are: Small-molecule receptors (blue), Neuropeptide receptors (green), Opsins (orange), LGRs (brown), Orphan receptors (gray). Colors representing decapod crustacean species are: *P*. *argus* (red), *H*. *americanus* (green), *P*. *clarkii* (pink), *C*. *sapidus* (blue). The model of substitution is LG+F+I+G4 according to BIC as selected by ModelFinder. Tree is unrooted and the root is drawn on the opsin clade. Scale bar represents expected number of substitutions per site. Ultrafast bootstrap support values are color-coded and size-scaled from 15 to 100.

Phylogenetic analyses of the sequences expressed in the transcriptomes of the four decapod species–*P*. *argus*, *H*. *americanus*, *P*. *clarkii*, *and C*. *sapidus*–revealed 333 sequences encoding putative class A GPCRs belonging to five subclasses: opsins, small-molecule receptors, neuropeptide receptors, leucine-rich repeat-containing GPCRs, and orphan receptors, (Figs 1–4). Differential expression values are shown in four supplemental tables: *P*. *argus*, S1 Table; *H*. *americanus*, S2 Table; *P*. *clarkii*, S3 Table; and *C*. *sapidus*, S4 Table. A summary of expression is shown in S5 Table. The five subclasses of class A GPCRs and their representatives in the decapod transcriptomes are described in the following sections.

### Opsins

We discovered 19 putative opsin sequences encoding eight different receptor types (Fig 1, S5 Table). We identified nine sequences encoding putative opsins generally found in the crustacean visual system [21], including six long wavelength-sensitive (LWS) opsins and three middle wavelength-sensitive (MWS) opsins in *P*. *argus*, *H*. *americanus*, and *P*. *clarkii*. We did not find any homologues for short wavelength-sensitive (SWS) opsins from crustaceans. We identified one homologue for *Drosophila* Rh7 in *C*. *sapidus*, and onychopsin-like sequences in *P*. *argus* and *C*. *sapidus*. Ultrafast bootstrap support is low (81) for the onychopsin cluster (Fig 1); however, when the NCBI non-redundant protein database (nr) is queried with the Parg_oncychopsin-like sequence, BLASTP reports the onychopsin sequence from the velvet worm, *Euperipatoides kanangrensis* as one of the top 5 hits (e-value: 2 e-90) with 41% shared identity. We also identified homologues for four non-visual opsins: pteropsin, peropsin, arthropsin, and neuropsin. Homologues for pteropsin and arthropsin were identified in *C*. *sapidus*, homologues for peropsin were identified in *H*. *americanus*, *P*. *clarkii*, and *C*. *sapidus*, and homologues for neuropsin were identified in *P*. *clarkii* and *C*. *sapidus*. None of the putative opsins identified in this study were conserved in all four crustaceans.

### Small-molecule receptors

There are two major neurotransmitter classes comprising peptides and small-molecule neurotransmitters. Small-molecule neurotransmitters comprise low molecular weight neurotransmitters such as biogenic amines, amino acids, purines, eicosanoids, and acetylcholine. Twenty-six putative small-molecule receptor subtypes were identified in the four decapods (Fig 2, S5 Table). Of these, 20 were biogenic amine receptors, including three putative dopamine receptors (D1⍺R, D1βR, D2⍺R), one dopamine/ecdysteroid receptor (DopEcR), six serotonin receptors (5HT1AR, 5HT1BR, 5HT2AR, 5HT2BR, 5HT4R, 5HT7R), six octopamine receptors (Oct⍺R, Oct⍺2AR, Octβ1R, Octβ2R, Octβ3R, Octβ4R), one α1-adrenergic receptor (⍺1AdrR), two tyramine receptors (TyrR1, TyrR2), and one histamine receptor (H1R). The other six small-molecule receptors include two muscarinic acetylcholine receptors (mAChR_A, mAChR_B), three eicosanoid receptors for prostaglandins (PGE2R1, PGE2R2, PGD2R), and one adenosine receptor (AdoR). Small-molecule receptors are highly conserved among the four decapods, with most being present in three or four of the species. Putative receptors expressed in all four species include TyrR1, 5HT7R, DopEcR, AdoR, 5HT4R, PGE2R2, Octβ2R, Octβ3R, and Octβ4R. Putative receptors conserved in the brain transcriptomes from *P*. *argus*, *H*. *americanus*, and *P*. *clarkii* include Oct⍺2AR, mAChR_A, D1βR, and D2⍺R.

### Neuropeptide receptors

Neuropeptide receptors were the most diverse receptor subclass in this study. We identified 44 subtypes with putative homologues for the following receptors: sulfakinin (SKR), natalisin (NTLR), tachykinin (TKR), RYamide (RYa-R), leucokinin (LKR), neuropeptide F (NPFR), long neuropeptide F (LNPFR), short neuropeptide F (SNPFR), SIFamide (SIFa-R), allatostatin A (AstA-R), allatostatin C (AstC-R), CCHamide (CCHa-R), proctolin (ProcR), myosuppressin (MSR), FMRFamide (FMRFa-R), CNMamide (CNMa-R), pyrokinin (PKR), capability (CapaR), ecdysis triggering hormone (ETH-R), trissin (TrisR), elevenin (ElevR), crustacean hyperglycemic hormone (CHH-R), adipokinetic hormone/corazonin-related peptide (ACP-R), red pigment concentrating hormone (RPCH-R), corazonin (CrzR), inotocin (InotR), and crustacean cardioactive peptide (CCAP-R) (Fig 3, S5 Table). We found five putative neuropeptide receptors in all four decapod crustaceans (ElevR1, FMRFa-R, CHH-R, AstA-R, CCAP-R) and three in all three brain transcriptomes (TKR, NPFR2, SNPFR) (S5 Table).

### Leucine rich repeat containing GPCRs

LGRs are defined by the large leucine-rich ectodomain, which serves as the ligand-binding region for glycoprotein hormones [70]. There are five known LGRs in arthropods: LGR1, LGR2, LGR3, LGR4, and LGR5 [71]. Annotations for LGRs correspond with *Drosophila* nomenclature described by Van Hiel et al. 2012 [71]. We identified two putative homologues each for LGR1 (LGR1-R1, LGR1-R2), LGR2 (LGR2-R1, LGR2-R2), and a single homologue for LGR3, LGR4, and LGR5 (Fig 3, S1–S4 Tables). LGR1-R2, LGR2-R1, and LGR2-R2 are expressed in all four decapods. LGR3 and LGR5 are conserved in the brain transcriptomes from *P*. *argus*, *H*. *americanus*, and *P*. *clarkii*, while a single homologue for LGR4 was found in *P*. *clarkii*.

### Orphan receptors

We identified 117 sequences encoding putative orphan receptor in the four decapod transcriptomes, including homologues for ten orphan receptors identified in prior studies in other species, though their ligands and/or functions are unclear. These include homologues for seven putative *Drosophila* orphan receptors (Moody, Tre1, CG4313, CG33639, CG13995, CG13579, CG12290), one orphan receptor previously identified in *P*. *clarkii* (HP1R), and two homologues for orphan receptors originally found in vertebrate species (GPR161, GPR150) (Fig 3, S5 Table). A number of sequences were classified as orphan receptors, as we could not determine their identity based on phylogeny or BLAST. Of the 117 putative orphan sequences, 83 are without a homologue in another species: 19 in *P*. *argus*, 20 in *H*. *americanus*, 23 in *P*. *clarkii*, and 21 in *C*. *sapidus* (Fig 4). There are some orphan receptor clusters that share homology with known subclasses. For example, the branch containing GPCR_L1-GPCR_L9 splits off the branch containing LGRs (Fig 4), and branching is supported by ultrafast bootstrap (93). We also found one orphan sequence that clusters with adenosine receptors (Csap_GPCR_M1), and one sequence that clusters with opsins (Pcla_GPCR_M1), though we were unable to find a functionally characterized reference homologue for these sequences (Fig 4).

### Receptor expression levels in chemosensory organs and brain

From the three decapods with transcriptomes from the brain and chemosensory organs (*P*. *argus*, *H*. *americanus*, *P*. *clarkii*), we identified 55 transcripts with higher relative expression (fold-change > 2.82) in the chemosensory organs than the brain (LF vs. Brain and Dactyl vs. Brain). Of these 55 transcripts, 40 have higher relative expression (fold-change > 2.82) in the LF than brain (10 in *P*. *argus*, 11 in *H*. *americanus*, and 19 in *P*. *clarkii*, Table 3), and 22 have higher expression in the dactyl than brain (9 in *P*. *argus*, 11 in *H*. *americanus*, and 2 in *P*. *clarkii*, Table 4) (S2 Fig).

**Table 3:** Class A GPCRs enriched in LF compared to brain.

Species	Transcripts of GPCRs enriched in LF			
	Opsins	Small-molecule	Neuropeptide	Orphan
*P*. *argus*	Onychopsin-like	TyrR1, H1R	CCHa-R1b	HP1R, GG13995, GPCR_E1, GPCR_G1, GPCR_I1, GPCR_K1
*H*. *americanus*	-	DopEcR, OctαR, TyrR1, H1R, PGE2R2b	ProcR1	HP1R, CG13995, GPCR_C2, GPCR_E1, GPCR_G1
*P*. *clarkii*	Neuropsin	PGE2R1, PGE2R2a	ACP-R1, RPCH-R2, CHH-Rb	MoodyR, Tre1R, CG12290, GPR150, GPCR_A3, GPCR_C3, GPCR_D2y, GPCR_E1, GPCR_F1, GPCR_F2y, GPCR_G1, GPCR_K1, GPCR_L9

Class A GPCR transcripts with higher relative expression (fold-change > 2.82) in LF based on DESeq2 analysis of the organ comparisons, LF vs. Brain.

**Table 4:** Class A GPCR enriched in dactyl compared to brain.

Species	Transcripts of GPCRs enriched in Dactyl			
	Opsins	Small-molecule	Neuropeptide	Orphan
*P*. *argus*	-	TyR2	CCHa-R1b, ACP-R1, RPCH-R2	GPCR_A1, GPCR_C2x, GPCR_E1, GPCR_G1, GPCR_K1
*H*. *americanus*	LWS_Opsin1, LWS_Opsin3	5HT4R, PGE2R2a, PGE2R2b, H1R	FMRFa-R	CG12290, GPCR_L4, GPCR_L5, GPCR_E1
*P*. *clarkii*	-	-	CHH-Rb	GPCR_A3

Class A GPCR transcripts with higher relative expression (fold-change > 2.82) in dactyls based on DESeq2 analysis of the organ comparisons, Dactyl vs. Brain.

There were eight transcripts expressed at higher levels (fold-change > 2.82) in both the LF and dactyl relative to the brain including CCHa-R1b, GPCR_E1, and GPCR_G1 in *P*. *argus*; H1R, PGE2R2b, and GPCR_E1 in *H*. *americanus;* and CHH-Rb and GPCR_A3 in *P*. *clarkii*. Several class A GPCR transcripts had higher fold differences in expression in chemosensory organs compared to the brain in each species: Orphan GPCR_E1 had the highest relative expression of any putative class A GPCR in the LF relative to the brain in *P*. *argus* (822-fold) and in *P*. *clarkii* (1674-fold). Parg_GPCR_E1 is also more highly expressed in the dactyl (346-fold) relative to the brain. Hame_GPCR_E1 was also more highly expressed in the LF (33-fold) relative to the brain; however, Hame_H1R had the highest relative expression of any class A GPCR transcript in the LF compared to the brain (64-fold). Parg_H1R was also more highly expressed in the LF (47-fold) compared to the brain. Another orphan transcript, GPCR_G1, also had higher expression in the LF compared to the brain in all three species at 62-fold relative to the brain in *P*. *argus*, 37-fold in *H*. *americanus*, and 9.3-fold in *P*. *clarkii*.

Expression patterns between LF and dactyl varied between species. The number of transcripts expressed at higher levels (fold-change > 2.82) in the LF than dactyl are: 17 in *P*. *argus*, 22 in *H*. *americanus*, 73 in *P*. *clarkii*, and 25 in *C*. *sapidus* (S1–S5 Tables). The number of transcripts expressed at higher levels (fold-change > 2.82) in the dactyl than LF are: 33 in *P*. *argus*, 17 in *H*. *americanus*, 5 in *P*. *clarkii*, and 6 in *C*. *sapidus* (S1–S5 Tables).

## Discussion

Our study identified dozens of class A GPCRs in two chemosensory organs (antennular lateral flagellum [LF] and walking leg dactyls, see S1 Fig) of four species of decapod crustaceans (Caribbean spiny lobster *Panulirus argus*, American lobster *Homarus americanus*, red-swamp crayfish *Procambarus clarkii*, and blue crab *Callinectes sapidus*), and brain (supraesophageal ganglion) of *P*. *argus*, *H*. *americanus*, and *P*. *clarkii*. We chose these species because they are among the major models of crustacean chemoreception [39–41]. These decapod GPCRs belong to five subclasses of class A GPCRs: opsins, small-molecule receptors, neuropeptide receptors, leucine-rich repeat-containing GPCRs, and orphan receptors. We identified novel sequences enriched in the LF including an onychopsin-like receptor, a metabotropic histamine receptor, and numerous orphan receptors. These receptors and their possible functions, including in chemical sensing, neuromodulation, and neurotransmission, are discussed below.

### Opsins

Opsins are used as visual pigments in ocular and extra-ocular photoreception in many animals, including crustaceans [62–65]. In the decapod species used in our study, we identified visual subtypes (LWS, MWS) in three extraocular organs–LF, dactyl, and brain–with the expression profiles varying across species and organ. These subtypes are known to transduce light at visible wavelengths and are expressed mainly in the retina, but have been found throughout the CNS of crustaceans [72–75]. The expression and function of visual opsins in the chemosensory organs of crustaceans is obscure. Opsins with non-visual functions are also known in animals. For example, opsins in the chemosensory organs of *D*. *melanogaster* mediate non-visual sensory modalities, including chemoreception [33], audition [76], and thermoreception [77]. An example is *Drosophila* Rh7 [78], which functions as a bitter taste receptor in the proboscis of fruit flies [33]. A homologue to *Drosophila* Rh7 is present in the LF and dactyl of *C*. *sapidus*, establishing it as a candidate chemoreceptor in decapods.

We also found an onychopsin-like sequence in *P*. *argus* and *C*. *sapidus* that is enriched in the LF, absent in the dactyl, and expressed at lower levels in the brain. This onychopsin-like sequence is expressed in olfactory sensory neurons of *P*. *argus* [41]. Onychopsin is the sole visual opsin in the Onychophora [79, 80], commonly known as velvet worms, which is the phylogenetic sister group to Arthropoda [81]. Onychopsin is the speculative origin that gave rise to the mass expansion of visual opsins in arthropoda [82]. Onychopsin is not known to be expressed in arthropods and we are uncertain of its function in the chemosensory organs of decapod crustaceans.

We also identified putative homologues for four non-visual opsins (neuropsin, arthropsin, peropsin, pteropsin) all of which are expressed in the decapod chemosensory organs. Functional characterization of non-visual opsins is lacking in crustaceans; however, there is evidence for their function in mammals. For example, neuropsin is a UV-light sensor in mammals [83, 84], this raises the possibility that it contributes to the UV-light sensitivity reported in decapod crustaceans [85]. Neuropsin is enriched in the LF of *P*. *clarkii* and may allow for UV-light detection in this sensory organ. While opsins are great candidates for sensory receptors in the chemosensory organs of decapod crustaceans; we can only speculate until functional analysis is performed for these putative receptors.

### Small-molecule receptors

Small-molecule receptors are highly conserved and expressed throughout the brain and chemosensory organs of the four decapods. Most of these small-molecule receptors are homologues to sequences from *D*. *melanogaster* and other crustaceans. However, they also include several unexpected homologues including putative homologues for an adrenergic receptor, histamine receptor, and one novel serotonin receptor. A putative homologue to a 5HT4R serotonin receptor was identified in three of the decapod species, and these are expressed in the brain and both chemosensory organs. These decapod 5HT4R sequences share homology with the 5HT4R of the sea cucumber *Apostichopus japonicus*, where it is highly expressed in its peripheral organs, especially the respiratory tree [86]. However, the decapod 5HT4R sequences do not contain the conserved N-R-F motif, but rather an H-R-F motif in the third transmembrane domain, which is distinct from all other small-molecule receptors (S6 File, S10 File). Although these transcripts share homology with serotonin receptors, the ligand and function of the receptors in decapods remain unclear.

Another novel receptor type was found in *H*. *americanus*: a homologue to the ⍺1-adrenergic receptor in the polychaete annelid *Platynereis dumerilii*. Bauknecht and colleagues [87] provide evidence for the coexistence of octopamine and adrenergic signaling systems in protostomes, and they functionally characterized ⍺1 and ⍺2 adrenergic receptors in *P*. *dumerilii*. Our findings suggest that adrenergic receptors may exist in crustaceans as well. This adrenergic receptor was expressed in the brain and absent from the chemosensory organs.

We also found expression of a metabotropic H1 histamine receptor (H1R) conserved in three decapod crustaceans. Ionotropic histamine-gated chloride channels have been physiologically characterized in the OSNs of decapod crustaceans [88], but our study is the first to document the expression of a metabotropic histamine receptor in crustaceans, or even in arthropods. H1R has yet to be functionally characterized in protostomes, and there is limited support for its existence in protostome or deuterostome invertebrates. H1R has been functionally characterized in only one invertebrate, the sea urchin *Strongylocentrotus purpuratus*. Leguia and Wessel [89] found H1R expressed on the surface of eggs of *S*. *purpuratus*, and they proposed that H1R influences fertilization by regulating nitric oxide production. Lutek and colleagues [90] show that H1R localizes to the mouth region during the larval stages, which may suggest involvement in chemical sensing. Decapod H1R sequences share homology with H1Rs in mammals including the conserved residues that define the histamine binding pocket (S6 File, S10 File) [91]. Decapod H1Rs are enriched in the LF of *P*. *argus*, *H*. *americanus*, and *C*. *sapidus*. In fact, *P*. *argus*, H1R is the most highly expressed small-molecule receptor in the LF and its OSNs [41], supporting the possibility that H1R plays a role in olfaction in decapods.

Several other small-molecule receptors are more highly expressed in the chemosensory organs than the brain, including the E2 prostaglandin receptor and receptors for tyramine and octopamine. Both tyramine and octopamine modulate olfactory processes, including pheromone sensitivity in arthropods [92–95]. Among the octopamine receptors, Octβ3R has the highest expression levels in the OSNs of *P*. *argus* [41]. Both tyramine receptor homologues have enriched expression in the LF: TyrR1 in *P*. *argus* and TyrR1 and TyrR2 in *H*. *americanus*. While only TyrR1 is expressed in OSNs of *P*. *arugs* [41]. There is little support for prostaglandins mediating chemical sensing, although we found PGE2R to be more highly expressed in both chemosensory organs than in the brain of the decapods. Only recently has PGE2R been functionally characterized in an arthropod, *M*. *sexta* [96]. Decapod PGE2Rs do not share homology with the *M*. *sexta* PGE2R, but they do share homology with vertebrate PGE2R.

The dopamine/ecdysteroid receptor is also expressed at higher levels in the LF compared the brain. DopEcR can bind 20-hydroxyecdysone (20E) and dopamine, but it more readily binds 20E [97]. Abrieux and colleagues [98] found that DopEcR modulates pheromone perception in the moth *Agrotis ipsilon* by binding 20E in the antennal lobe. In *D*. *melanogaster*, DopEcR is co-expressed in GR5a gustatory neurons and is activated by dopamine, which enhances sugar sensitivity [99]. Our findings also suggest that DopEcR may possibly modulate chemical sensing in decapods since DopEcR is expressed in the LF and OSNs of *P*. *argus* [41].

Two serotonin receptors were detected in the OSNs of *P*. *argus*: 5HT1AR and 5HT7R [41]; however, expression is higher in the brain compared to the LF and dactyl for both transcripts. Similar to *D*. *melanogaster*, 5HT1AR is expressed in OSNs expressing variant ionotropic receptors, but unlike *D*. *melanogaster*, 5HT2BR is not expressed in OSNs of *P*. *argus* [41, 100]. While 5HT2BR is absent from the OSN transcriptomes of *P*. *argus*, 5HT7R is expressed in them, suggesting a similar mechanism of serotonergic modulation of OSNs involving two distinct serotonin receptor subtypes.

Several small-molecule receptors are highly expressed in OSNs of *D*. *melanogaster* that express either Olfactory Receptors (ORs) or variant Ionotropic Receptors (Irs), including the dopamine receptor Dop1R1, DopEcR, mAChR-B, and two octopamine β-receptors [100]. Similar to *D*. *melanogaster*, DopEcR, mAChR-B, and two octopamine β-subtypes are expressed in the OSNs of *P*. *argus* [41]. Expression of biogenic amine receptors in chemosensory organs and OSNs of crustaceans and insects appears to be very similar. However, there is one key difference: homologues to the metabotropic histamine receptor H1R were found in crustaceans but not insects or chelicerates, suggesting that H1R may be a crustacean-specific receptor within the Arthropoda.

#### Neuropeptide receptors

Many (44) neuropeptide receptors were identified in our study, most having higher expression in the brain, though several have higher expression in the chemosensory organs. For example, ACP-R and the RPCH-R have varied expression in the chemosensory organs between species. ACP-R1 and RPCH-R2 are more highly expressed in the LF of *P*. *clarkii*, but are more highly expressed in the dactyl of *P*. *argus*. The function of ACP receptors remains unclear but they have been implicated in regulating ecdysis in insects [101–103]. RPCH-R has been shown to be expressed at high levels in the dactyl and other appendages [104], and it functions to concentrate red pigment in the chromatophores [105]. The FMRFamide receptor and CHH receptor also demonstrate high expression levels in the dactyl. FMRFa-R is enriched in the dactyl of *H*. *americanus* and CHH-Rb is enriched in the dactyl of *P*. *clarkii*. FMRFamide is a putative neurotransmitter for chemosensory neurons in the dactyl [106]. Our findings support this idea, since FMRFa-R expression is elevated in the dactyl of *H*. *americanus*. CHH-Rb is also the most highly expressed putative class A GPCR in the dactyl of *P*. *clarkii*. The CHH receptor has yet to be functionally characterized in crustaceans, though its expression is associated with molt cycle regulation [18]. Two CHH receptors (BNGR-A34 and BNGR-A2) have been functionally characterized in the silk moth *B*. *mori*. Sun et al. (2020) [107] reported that BNGR-A2 maintains osmotic balance and supports gut motility. However, decapod sequences share homology with BNGR-A34, and little is known about the function of BNGR-A34 in *B*. *mori* except that it is highly expressed in somatic tissue and has low expression in neural tissue.

Overall, expression of neuropeptide receptors is lower in the LF compared to other subclasses, with the exception of the proctolin receptor in *H*. *americanus*, which has the highest expected count of any putative class A GPCRs in the LF (S2 Table). The role of proctolin in the LF is unclear, though it is a cardioacceleratory neuropeptide in arthropods that regulates the function of the auxiliary heart and circulation of hemolymph [108, 109].

Neuropeptide receptor expression in the OSNs of the LF is sparse, with only the allatostatin C receptor (AstCR2) expressed in the OSNs of *P*. *argus* [41]. Though AstCR2 was broadly expressed in OSNs of *P*. *argus*, its expression in LF is low compared to the brain. Liu et al. [110] reported that AstC neuropeptide is expressed in the olfactory lobe of decapod crustaceans. The function of the AstC signaling in decapod OSNs and olfactory lobes is unclear, but AstC has been shown to modulate olfactory learning and pheromone sensitivity in insects [111], and influences circadian rhythm and immunity [112, 113].

Based on these studies, we hypothesize that in the LF of decapod crustaceans, several neuropeptide receptors, including ACP-R, RPCH-R, and the proctolin receptor, may support homeostatic functions, and AstCR2 may modulate the sensitivity of OSNs. CHH-R and FMRFa-R may support homeostatic function or modulate chemoreception in the dactyl. While there are several neuropeptide receptors enriched in the chemosensory organs; expression patterns suggest that neuropeptide receptors dominate the brain and are likely more pertinent to CNS function.

#### Leucine-rich repeat-containing GPCRs

LGRs are found throughout the central and peripheral nervous system of decapod crustaceans. LGRs act as receptors for glycoprotein hormones and carry out numerous functions. LGR3, LGR4, and LGR5 serve as receptors for gonadulin, relaxin, and arthropod insulin-like growth factor respectively [114]. In *D*. *melanogaster*, centrally expressed LGR3 regulates growth and coordinates developmental timing [115–117]. In decapods, LGR3 is also enriched in the brain and may serve a similar function as it does in insects. LGR4 and LGR5 are also enriched in the brains of the decapods, but there is little information on the function of LGR4 and LGR5 in arthropods. The function of LGR1 and LGR2 is well described in insects. For example, LGR2 serves as the receptor for the glycoprotein hormone bursicon, which regulates hardening, tanning, and molting of the cuticle in insects and crustaceans [118–120]. LGR2 also regulates wing-expansion behavior in insects, which is stimulated by the circulation of hemolymph [121]. In crustaceans, unlike most LGR receptors that are more highly expressed the brain, LGR2 has similar expression in the brain and chemosensory organs. LGR1 is also implicated in mediating multiple functions in insects. For example, LGR1 serves as a receptor for the heterodimeric glycoprotein hormone GPA2/GPB5, and it regulates spermatogenesis [122]. However, there is a larger body of evidence demonstrating that LGR1 is critically involved in regulating osmotic balance [123, 124]. Several studies also report high expression levels of LGR1 in antennae of insects [125, 126]. Likewise, in decapod crustaceans, LGR1 is highly expressed in the LF and its OSNs [41], though its expression is not restricted to the LF. We hypothesize that as in insects, LGR1 regulates osmolarity in the neurons of crustaceans.

In summary, in decapod crustaceans, LGR1 and LGR2 are more evenly expressed throughout the LF, dactyl, and brain, while LGR3, LGR4, and LGR5 are more highly expressed in the brain compared to the chemosensory organs. Of the five LGRs, only LGR1 is expressed in the OSNs of *P*. *argus*, raising the possibility that it may modulate OSN function in response to circulating GPA2/GPB5.

#### Orphan receptors

Homologues to seven *Drosophila* orphan receptors were identified, including: CG4313, CG12290, CG13995, CG33639, CG13579, Tre1, and moody receptor. Previous studies suggest that CG4313, CG12290, CG13995, and CG33639 are orphan neuropeptide receptors [15, 127], while CG13579 is similar to DopEcR, and is a suspected variant ecdysteroid receptor [128]. The predicated classes are speculative since the ligands and functions are unknown. While little is known about their function, homologues for CG13995 and CG13579 are expressed in OSNs of *P*. *argus* [41], which is consist with a role in chemical sensing. Unlike the aforementioned receptors, the functions of moody and tre1 have been described in numerous studies. Moody receptors are expressed on the surface of glial cells in the CNS and regulate the formation and permeability of the blood brain barrier [129, 130]. Moody receptors are expressed in the brain and chemosensory organs of the decapod crustaceans. Moody receptors may have multiple functions especially in the chemosensory organs; however, they are unlikely to be directly involved in chemical sensing since they were not detected in OSNs of *P*. *argus* [41]. Tre1 receptors have been implicated in chemical sensing and in regulating male courtship behavior in *Drosophila* showing sexually dimorphic expression in OSNs [131]. We did find Tre1R to be enriched in the LF of *P*. *clarkii*; however, Tre1R was not expressed in the OSNs of *P*. *argus* [41]. Tre1 receptors also regulate migration of germ cells and immune cells in *Drosophila* [132, 133], so non-sensory functions in crustaceans are possible.

We also identified one crustacean orphan receptor known as HP1R. HP1R was originally discovered in the hepatopancreas of *P*. *clarkii* [134]. Susceptibility and immunologic response to pathogenic bacteria, such as *Aeromonas hydrophila*, are associated with expression of HP1R in *P*. *clarkii* [135], although the specific ligand of this receptor remains in question. HP1R is expressed in the LF of *P*. *argus* and *H*. *americanus*, and is expressed in OSNs of *P*. *argus* [41]. The endogenous ligand of HP1R is unclear, one possibility is that it detects metabolic byproducts produced by pathogenic bacteria such as the volatile fatty acids produced by *A*. *hydrophila* [136]. A mechanism to detect harmful microbes infiltrating the LF would be advantageous since many species of bacteria can colonize the LF [137].

Two other putative orphan receptors identified in this study–GPR150 and GPR161 –share homology with orphan receptors classified in vertebrates. Little is known about their phylogeny or function. In the phylogenetic tree, GPR150 clusters closely with inotocin receptors indicating that these transcripts have some shared homology. GPR161 clusters with the *Drosophila* orphan receptor CG12290; however, unlike CG12290, GPR161 is expressed in the LF and OSNs of *P*. *argus* [41] suggesting a possible involvement with chemical sensing.

Several other orphan receptors are expressed at higher levels in the chemosensory organs, mainly in the LF. GPCR_E1 and GPCR_G1 were more highly expressed in the LF than brain of *P*. *argus*, *H*. *americanus*, and *P*. *clarkii*, and both transcripts were expressed in the OSNs of the LF from *P*. *argus* [41]. Additionally, GPCR_C1, GPCR_G2, GPCR_G2, GPCR_I1, and GPCR_L2 were expressed in the OSNs of *P*. *argus* [41]. Although their function and ligand selectivity are not clear, these receptors are candidate chemoreceptors or neuromodulators in the OSNs of crustaceans. In summary, the function of these orphan receptors is difficult to discern at present. Functional testing is needed to determine their ligand specificity and activation. Nonetheless, their expression in the chemosensory organs and OSNs of decapod crustaceans suggest that some may play a role in chemical sensing.

## Conclusions

Class A GPCRs are expressed in the chemosensory organs and brain of decapod crustaceans. We identified a subset of class A GPCRs that are enriched in the LF and dactyl. These receptors may mediate chemical sensing, possibly olfaction, though their roles remain undetermined. We can only speculate the function and the expression of these putative receptors. mRNA can be used as a measure to predict protein expression; however, mRNA may not directly translate to protein expression. We can only infer that transcripts expressed more highly in a particular organ may be important for its function. Analysis of the proteome and functional characterization of these transcripts will be essential in deciphering the role of these putative receptors in decapod crustaceans.

## Supporting information

S1 Text Predicted protein sequences for all GPCRs in this study. (TXT)

S1 Fig Overview of spiny lobster chemosensory systems. **(a)** Location of aesthetascs mediating olfaction (blue dots) and bimodal chemo- and mechanosensory sensilla mediating distributed chemoreception (yellow dots) on different body parts and appendages of *P*. *argus* (1—lateral flagellum of antennule, 2—medial flagellum of antennule, 3—second antenna, 4—mouthpart appendages, 5—walking legs, 6—gill chamber, 7—tail fan, 8—pleopods). Location of pieces of appendages used for immunocytochemistry and PCR indicated by gray boxes: dactyl, 2^nd^ antenna (A2). **(b)** Location of aesthetascs and bimodal chemo- and mechanosensory sensilla on the antennules. Aesthetascs (blue) are restricted to a tuft of sensilla on the distal third of the lateral flagellum. Bimodal chemo- and mechanosensory sensilla (yellow) among them, guard setae (GS) are associated with the aesthetascs but also occur on the proximal part of the lateral flagellum and on the entire medial flagellum. Location of pieces of appendages used for immunocytochemistry and PCR indicated by gray boxes: lateral flagellum of antennule proximal (LFP), lateral flagellum of antennule distal (LFD). **(c)** Schematic drawing of the cellular organization of olfactory sensilla. Olfactory sensilla, called aesthetascs, are exclusively innervated by olfactory receptor neurons (ORN, blue). The outer dendritic segments of the ORNs (modified cilia) are highly branched and covered by extremely thin and permeable cuticle (pC). **(d)** Schematic drawing of the cellular organization of distributed chemosensilla. Distributed chemosensilla are bimodal chemo- and mechanosensory sensilla innervated by a few mechanoreceptor neurons (MRN) and several chemoreceptor neurons (CRN). Dendrites of CRNs are unbranched and extend to a terminal pore (tP) at the tip of the sensillum. Adapted from [39].
(TIF)

S2 Fig MA-plots of DESeq2 analyses between organ types of four decapod crustaceans. MA-plots showing distribution of transcripts from DESeq2 analyses between organ types. Each grey dot represents a transcript from the transcriptomes of Parg (*Panulirus argus*), Hame (*Homarus americanus*), Pcla (*Procambarus clarkia*), and Csap (*Callinectes sapidus*). Red and green threshold lines indicate Log_2_ FC of ±1.5. Distribution of transcripts for five different class A GPCR subclasses (opsin, small-molecule, neuropeptide, LGR, and orphan) are colored as indicated in the inset of each plot. DESeq2 analyses were gathered from Kozma et al. 2020a [Ref 40]. **(a)** LF vs. Da: Four MA-plots from DESeq2 analysis between LF and dactyl for each species. **(b)** LF vs. Br: Three MA-plots from DESeq2 analysis between LF and brain for each species. **(c)** LF vs. Da: Three MA-plots from DESeq2 analysis between dactyl and brain for each species.
(PDF)

S1 Table Expected counts matrix from RSEM analysis and fold changes from DESeq2 analysis for three organs (LF, dactyl, brain) from the Caribbean spiny lobster *Panulirus argus*. Receptors are organized by subclass: opsins, small-molecule receptors, neuropeptide receptors, leucine-rich repeat-containing GPCRs, and orphan receptors. Values are (from left to right) number of transcript expected counts from RSEM for each transcriptome, and the log_2_ fold change and actual fold change for LF vs. brain, LF vs. dactyl, and dactyl vs brain. Green box (RSEM expected count > 1000). Orange box (1000 > RSEM expected count > 100). Blue box = log_2_ FC > 1.5 between the two organs. Pink box = log_2_ FC < -1.5 between the two organs. Yellow box = log_2_ FC between –1.5 and 1.5.
(XLSX)

S2 Table Expected counts matrix from RSEM analysis and fold changes from DESeq2 analysis for three organs (LF, dactyl, brain) from the American lobster *Homarus americanus*. Receptors are organized by type: opsins, small-molecule receptors, neuropeptide receptors, leucine-rich repeat-containing GPCRs, and orphan receptors. Values are (from left to right) number of transcript expected counts from RSEM for each transcriptome, and the log_2_ fold change and actual fold change for LF vs. brain, LF vs. dactyl, and dactyl vs brain. Green box (RSEM expected count > 1000). Orange box (1000 > RSEM expected count > 100). Blue box = log_2_ FC > 1.5 between the two organs. Pink box = log_2_ FC < -1.5 between the two organs. Yellow box = log_2_ FC between –1.5 and 1.5.
(XLSX)

S3 Table Expected counts matrix from RSEM analysis and fold changes from DESeq2 analysis for three organs (LF, dactyl, brain) from the red swamp crayfish *Procambarus clarkii*. Receptors are organized by type: opsins, small-molecule receptors, neuropeptide receptors, leucine-rich repeat-containing GPCRs, and orphan receptors. Values are (from left to right) number of transcript expected counts from RSEM for each transcriptome, and the log_2_ fold change and actual fold change for LF vs. brain, LF vs. dactyl, and dactyl vs brain. Green box (RSEM expected count > 1000). Orange box (1000 > RSEM expected count > 100). Blue box = log_2_ FC > 1.5 between the two organs. Pink box = log_2_ FC < -1.5 between the two organs. Yellow box = log_2_ FC between –1.5 and 1.5.
(XLSX)

S4 Table Expected counts matrix from RSEM analysis and fold changes from DESeq2 analysis for two organs (LF, dactyl) from the blue crab, *Callinectes sapidus*. Receptors are organized by type: opsins, small-molecule receptors, neuropeptide receptors, leucine-rich repeat-containing GPCRs, and orphan receptors. Values are (from left to right) number of transcript expected counts from RSEM for each transcriptome, and the log_2_ fold change and actual fold change for LF vs. brain, LF vs. dactyl, and dactyl vs brain. Green box (RSEM expected count > 1000). Orange box (1000 > RSEM expected count > 100). Blue box = log_2_ FC > 1.5 between the two organs. Pink box = log_2_ FC < -1.5 between the two organs. Yellow box = log_2_ FC between –1.5 and 1.5.
(XLSX)

S5 Table Conserved GPCRs in transcriptomes of four decapod species. GPCR homologues are listed along with the species (*P*. *argus*, *H*. *americanus*, *P*. *clarkii*, *C*. *sapidus*) and organs (LF, dactyl, brain) in which they are expressed. ✅ = differentially expressed transcripts (FC > 2.8); organ of highest expression. Light blue box = transcripts not expressed in a species (not in any organ). Red box = transcripts not expressed in that organ.
(XLSX)

S6 Table Receptors annotation changes. Listed are the original names in Kozma et al. (2020b) [Ref 41] and the new names in this paper.
(XLSX)

S1 File Sequences for Fig 1. (FA)

S2 File Sequences for Fig 2. (FA)

S3 File Sequences for Fig 3. (FA)

S4 File Sequences for Fig 4. (FA)

S5 File Mafft aligned sequences for Fig 1. (FA)

S6 File Mafft aligned sequences for Fig 2. (FA)

S7 File Mafft aligned sequences for Fig 3. (FA)

S8 File Mafft aligned sequences for Fig 4. (FA)

S9 File Trimmed-Mafft aligned sequences for Fig 1. (FA)

S10 File Trimmed-Mafft aligned sequences for Fig 2. (FA)

S11 File Trimmed-Mafft aligned sequences for Fig 3. (FA)

S12 File Trimmed-Mafft aligned sequences for Fig 4. (FA)

## Abbreviations

GPCR: G protein-coupled receptor
IR: variant Ionotropic Receptor
LF: lateral flagella of the antennule
OSN: olfactory sensory neuron
