# Supplementary figures and images for: G protein-coupled receptors as candidates for modulation and activation of the chemical senses in decapod crustaceans
Source: PLoS One. 2021 Jun 4;16(6):e0252066. doi: 10.1371/journal.pone.0252066 (PMC8177520; doi:10.1371/journal.pone.0252066)

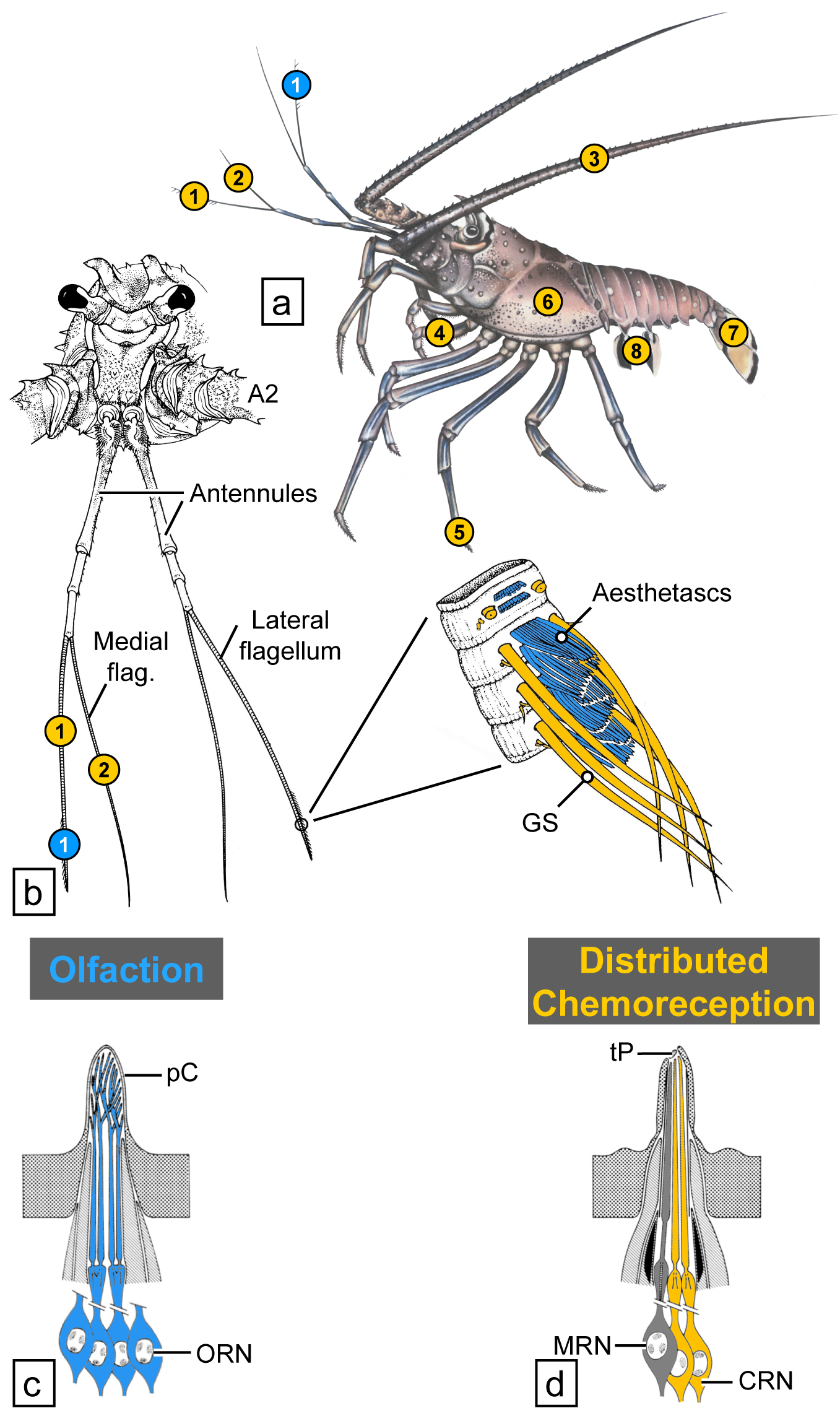

Supplement: S1 Fig — (a) Location of aesthetascs mediating olfaction (blue dots) and bimodal chemo- and mechanosensory sensilla mediating distributed chemoreception (yellow dots) on different body parts and appendages of P. argus (1—lateral flagellum of antennule, 2—medial flagellum of antennule, 3—second antenna, 4—mouthpart appendages, 5—walking legs, 6—gill chamber, 7—tail fan, 8—pleopods). Location of pieces of appendages used for immunocytochemistry and PCR indicated by gray boxes: dactyl, 2nd antenna (A2). (b) Location of aesthetascs and bimodal chemo- and mechanosensory sensilla on the antennules. Aesthetascs (blue) are restricted to a tuft of sensilla on the distal third of the lateral flagellum. Bimodal chemo- and mechanosensory sensilla (yellow) among them, guard setae (GS) are associated with the aesthetascs but also occur on the proximal part of the lateral flagellum and on the entire medial flagellum. Location of pieces of appendages used for immunocytochemistry and PCR indicated by gray boxes: lateral flagellum of antennule proximal (LFP), lateral flagellum of antennule distal (LFD). (c) Schematic drawing of the cellular organization of olfactory sensilla. Olfactory sensilla, called aesthetascs, are exclusively innervated by olfactory receptor neurons (ORN, blue). The outer dendritic segments of the ORNs (modified cilia) are highly branched and covered by extremely thin and permeable cuticle (pC). (d) Schematic drawing of the cellular organization of distributed chemosensilla. Distributed chemosensilla are bimodal chemo- and mechanosensory sensilla innervated by a few mechanoreceptor neurons (MRN) and several chemoreceptor neurons (CRN). Dendrites of CRNs are unbranched and extend to a terminal pore (tP) at the tip of the sensillum. Adapted from [39]. (TIF) [file pone.0252066.s002.tif]
