# Supplementary material for: G protein-coupled receptors as candidates for modulation and activation of the chemical senses in decapod crustaceans
Source: PLoS One. 2021 Jun 4;16(6):e0252066. doi: 10.1371/journal.pone.0252066 (PMC8177520; doi:10.1371/journal.pone.0252066)
Supplement: S2 Fig — MA-plots showing distribution of transcripts from DESeq2 analyses between organ types. Each grey dot represents a transcript from the transcriptomes of Parg (Panulirus argus), Hame (Homarus americanus), Pcla (Procambarus clarkia), and Csap (Callinectes sapidus). Red and green threshold lines indicate Log2 FC of ±1.5. Distribution of transcripts for five different class A GPCR subclasses (opsin, small-molecule, neuropeptide, LGR, and orphan) are colored as indicated in the inset of each plot. DESeq2 analyses were gathered from Kozma et al. 2020a [Ref 40]. (a) LF vs. Da: Four MA-plots from DESeq2 analysis between LF and dactyl for each species. (b) LF vs. Br: Three MA-plots from DESeq2 analysis between LF and brain for each species. (c) LF vs. Da: Three MA-plots from DESeq2 analysis between dactyl and brain for each species. (PDF) [file pone.0252066.s003.pdf]

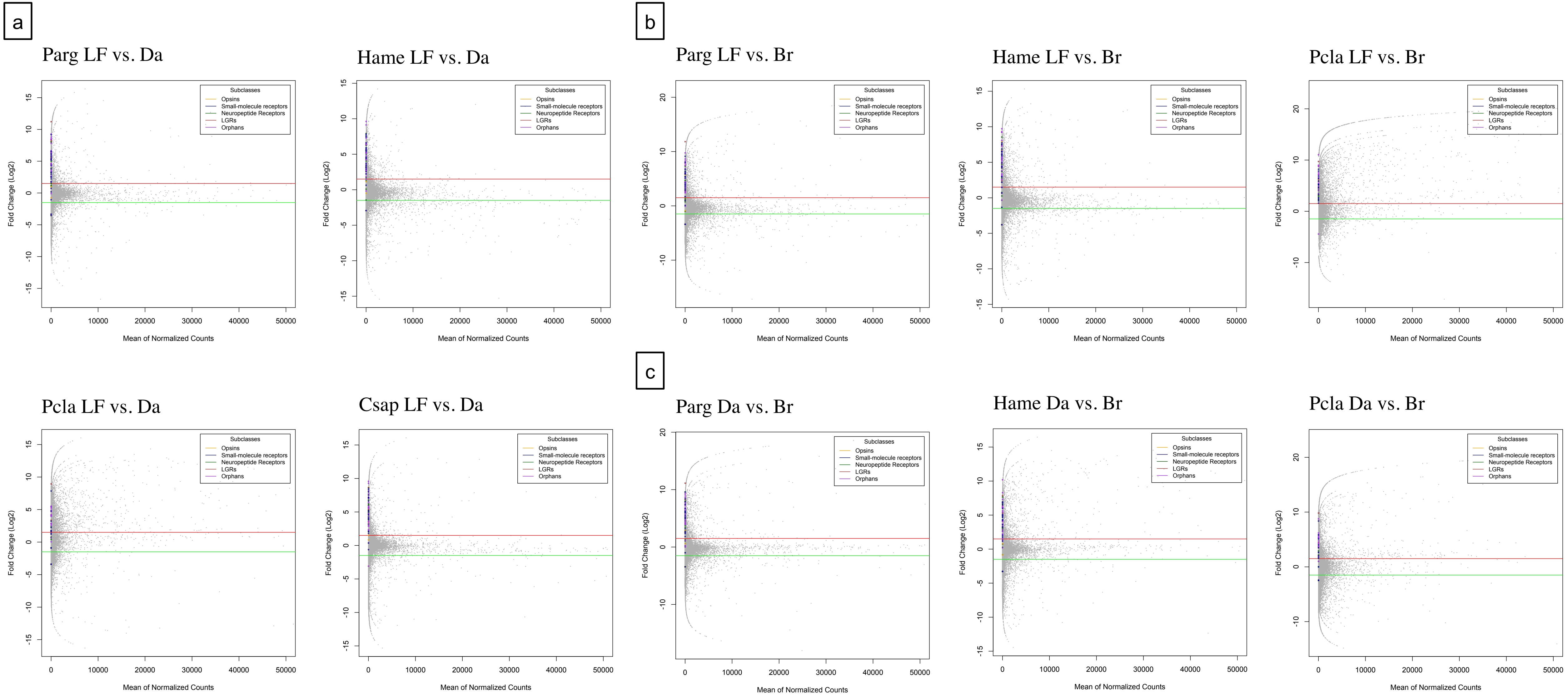
a
Parg LF vs. Da
Hame LF vs. Da
Pcla LF vs. Da
Csap LF vs. Da
b
Parg LF vs. Br
Hame LF vs. Br
Pcla LF vs. Br
c
Parg Da vs. Br
Hame Da vs. Br
Pcla Da vs. Br
Subclasses
Opsins
Small-molecule receptors
Neuropeptide Receptors
LGRs
Orphans
Fold Change (Log2)
Mean of Normalized Counts
